# A Novel Truncated Form of Serum Amyloid A in Kawasaki Disease

**DOI:** 10.1371/journal.pone.0157024

**Published:** 2016-06-06

**Authors:** John C. Whitin, Tom To-Sang Yu, Xuefeng Bruce Ling, John T. Kanegaye, Jane C. Burns, Harvey J. Cohen

**Affiliations:** 1 Department of Pediatrics, Stanford University, Stanford, California, United States of America; 2 Department of Surgery, Stanford University, Stanford, California, United States of America; 3 Department of Pediatrics, University of California San Diego, La Jolla, California, United States of America, and Rady Children’s Hospital–San Diego, San Diego, California, United States of America; University of Glasgow, UNITED KINGDOM

## Abstract

**Background:**

Kawasaki disease (KD) is an acute vasculitis in children that can cause coronary artery abnormalities. Its diagnosis is challenging, and many cytokines, chemokines, acute phase reactants, and growth factors have failed evaluation as specific biomarkers to distinguish KD from other febrile illnesses. We performed protein profiling, comparing plasma from children with KD with febrile control (FC) subjects to determine if there were specific proteins or peptides that could distinguish the two clinical states.

**Materials and Methods:**

Plasma from three independent cohorts from the blood of 68 KD and 61 FC subjects was fractionated by anion exchange chromatography, followed by surface-enhanced laser desorption ionization (SELDI) mass spectrometry of the fractions. The mass spectra of KD and FC plasma samples were analyzed for peaks that were statistically significantly different.

**Results:**

A mass spectrometry peak with a mass of 7,860 Da had high intensity in acute KD subjects compared to subacute KD (*p* = 0.0003) and FC (*p* = 7.9 x 10^−10^) subjects. We identified this peak as a novel truncated form of serum amyloid A with N-terminal at Lys-34 of the circulating form and validated its identity using a hybrid mass spectrum immunoassay technique. The truncated form of serum amyloid A was present in plasma of KD subjects when blood was collected in tubes containing protease inhibitors. This peak disappeared when the patients were examined after their symptoms resolved. Intensities of this peptide did not correlate with KD-associated laboratory values or with other mass spectrum peaks from the plasma of these KD subjects.

**Conclusions:**

Using SELDI mass spectrometry, we have discovered a novel truncated form of serum amyloid A that is elevated in the plasma of KD when compared with FC subjects. Future studies will evaluate its relevance as a diagnostic biomarker and its potential role in the pathophysiology of KD.

## Introduction

Kawasaki disease (KD), first described in 1967 [1], is an acute, self-limiting vasculitis of unknown etiology that usually occurs in infants and young children [2–5]. KD has replaced rheumatic fever as the leading cause of acquired heart disease in children in the US and other developed countries [6]. Coronary artery aneurysms or ectasia develop in 25% of untreated children with KD, and may lead to myocardial infarction, sudden death, or ischemic heart disease [7,8]. However treatment with intravenous immunoglobulin (IVIG) within the first ten days after fever onset reduces the risk of aneurysms to 5% [9].

There currently is no specific diagnostic test for KD, and diagnosis is often delayed [10,11]. Many classes of serum proteins are elevated in acute KD, including cytokines, chemokines, growth factors, and acute phase reactants such as C-reactive protein and serum amyloid A (SAA) [12–19]. However none reliably distinguishes children with KD from children with other febrile illnesses, which is the diagnostic dilemma confronting clinicians. We used mass spectrometry to discover potential new biomarkers and found higher levels of a truncated form of serum amyloid A (SAA) in the plasma of KD subjects when compared with FC subjects. Furthermore, levels of this fragment decreased when children recovered from the acute phase of KD.

## Materials and Methods

### Ethics

This study was approved by the Institutional Review Boards of the University of California, San Diego (UCSD) and Stanford University. Signed informed consent was obtained from all the subjects, and child or adolescent assent was obtained as appropriate. We received signed consent from the next of kin, caretakers, or guardians on behalf of the minors/children enrolled in our study. The protocol was approved by the Institutional Review Board at UCSD and covered the use of all clinical data and the sampling of extra blood.

### Study Subjects

Blood samples were collected from KD and febrile control (FC) subjects at Rady Children’s Hospital–San Diego. The diagnosis of KD was validated by JCB at the KD Research Center in UCSD according to an established protocol with standardized, prospective data collection. KD subjects met the American Heart Association criteria for KD [20]. Age-similar FC subjects were recruited from the Emergency Department if they had ≥3 days of fever and at least one of the clinical criteria for KD: rash, conjunctival injection, oral mucosa changes, extremity changes, and enlarged cervical lymph node. FC subjects with predominantly gastrointestinal or respiratory symptoms were excluded. Clinical data included age, sex, and illness day at diagnosis, and the following pre-treatment laboratory values are provided in Table 1: C-reactive protein (CRP), erythrocyte sedimentation rate (ESR), γ-glutamyl transpeptidase (GGT), platelet count, and white blood cell count (WBC). Plasma from three non-overlapping cohorts was used. All acute plasma samples from KD subjects were obtained prior to treatment with IVIG. A discovery cohort of 10 KD and 10 FC subjects was analyzed to identify the candidate mass spectrum peak, and to determine the nature of the corresponding protein. The second confirmation cohort of 43 KD and 42 FC subjects was analyzed to confirm the peptide peak was significantly elevated in KD compared to FC subjects. Additional subacute blood samples were obtained from 17 of the 43 KD subjects of the second confirmation study during the subacute phase of illness (KDsa, days 12–25 of illness). These patients received IVIG at the time of diagnosis and their clinical signs of KD had resolved. A third validation cohort of 15 KD and 9 FC subjects was used to validate the identity of the peptide using a hybrid mass spectrum immunoassay, and to determine if the peptide was the result of *ex vivo* proteolysis. Values for all clinical laboratory tests are not available for some subjects (Table 1).

**Table 1:** Demographic and laboratory characteristics of study subjects.

Discovery cohort	FC	n	KD	n	*p–*value^c^
Age^a^, years	1.22 (0.77, 2.99)	10	4.49 (1.61, 6.10)	10	0.063
Illness day at study^b^	5.4 (1.6)	10	6.6 (1.3)		NS
Sex^d^	5 M, 5 F		8 M, 2 F		NS^d^
IVIG resistant, *n*(%)	NA		2(20)		NA
Coronary artery status, *n* (%)					
Normal			7(70)		
Dilated			1(10)		
Aneurysm			2(20)		
CRP^a^, mg/dL	1.90 (0.73, 3.30)	4	11.10 (3.65, 18.40)	9	0.034
ESR^a^, mm/hr	31.5 (13.23, 62.23)	8	63.0 (56.5, 83.5)	10	0.025
GGT^a^, IU/L	19.0 (16.0, 20.0)	7	75.5 (63.8, 124.0)	10	0.006
Platelet count^a^, x10^3^/mm^3^	290 (258, 423)	10	440 (354, 519)	10	0.036
WBC count^a^, x10^3^/mm^3^	7.4 (6.2, 11.8)	10	12.1 (10.2, 16.1)	10	0.012
**Confirmation cohort**					
Age^a^	1.68 (0.93, 4.45)	42	2.43 (1.16, 4.06)	43	NS
Illness day at study^b^	4.6 (3.2)	42	7.7 (4.3)	43	< 1 x 10^−4^
Sex^d^	21 M, 21 F		26 M, 17 F		NS^d^
IVIG resistant, *n*(%)	NA		10(23)		NA
Coronary artery status, *n* (%)					
Normal			30(70)		
Dilated			2(5)		
Aneurysm			9(21)		
CRP	1.70 (0.58, 1.80)	30	6.10 (4.90, 12.90)	43	< 1 x 10^−4^
ESR	20.0 (13.8, 35.8)	26	59.0 (36.0, 75.0)	43	< 1 x 10^−4^
GGT	14.0 (12.0, 23.0)	24	31.0 (15.0, 122.0)	41	0.002
Platelet count	255 (179, 360)	39	425 (325, 606)	43	< 1 x 10^−4^
WBC count	8.9 (5.8, 13.4)	39	12.5 (10.7, 15.6)	43	5 x 10^−4^
**Validation and protease inhibitor cohort**					
Age^a^	4.42 (2.12, 5.88)	9	2.69 (2.12, 8.07)	15	NS
Illness day at study^b^	5.1 (1.8)	9	7.7 (4.4)	15	0.077
Sex^d^	6 M, 3 F		9 M, 6 F		NS^d^
IVIG resistant, *n*(%)	NA		1(7)		NA
Coronary artery status, *n* (%)					
Normal			14(93)		
Dilated			1(7)		
Aneurysm			0(0)		
CRP	1.50 (0.50, 3.45)	8	6.45 (4.03, 15.08)	14	0.019
ESR	23.0 (8.0, 36.0)	9	51.0 (40.0, 74.0)	15	0.002
GGT	20.0 (18.3, 38.0)	8	24.5 (17.8, 78.5)	14	NS
Platelet count	268 (174, 323)	9	372 (288, 508)	15	0.021
WBC count	6.4 (5.9, 11.7)	9	12.0 (9.7, 14.6)	15	0.017

Values for all clinical laboratory tests are not available for some subjects.

^a^Median (interquartile range (IQR))

^b^Mean (SD)

^c^Mann-Whitney *U* test; NS indicates *p* >0.1.

^d^Fisher’s Exact test; NS indicates *p* >0.1.

Coronary artery (CA) status of the KD subjects was assessed by echocardiography during the acute and subacute phase. Measurements of the internal diameters of the proximal right CA (RCA) and left anterior descending (LAD) CA were normalized for body surface area and expressed as standard deviation units from the mean (Z scores). CA abnormalities (CAA) were defined as follows: normal: Z score <2.5; dilated: ≥2.5 and < 4.0; aneurysm: Z score ≥4.0. The worst-ever Z score (Z worst) for either the RCA or LAD at any time point or the Z score of the largest aneurysm was used for the continuous variable analysis. IVIG resistance was defined as persistent or recrudescent fever (rectal or oral T ≥ 38.0°C) at least 36h post-end of IVIG infusion.

### Analysis of Samples

#### Samples

We collected blood in EDTA-containing tubes for all three cohorts. In Cohort 3, additional samples were collected in tubes containing EDTA and a broad spectrum of protease inhibitors (P100 v.2 tubes, Becton Dickinson). All samples were centrifuged, and the plasma supernatant was collected, aliquoted, and stored at -80°C until analysis.

#### Fractionation of plasma

Plasma samples for each cohort were processed in batches, as described [21,22]. Plasma (20 μL) was pretreated on ice for 30 min with 30 μL 9 M urea, 2%CHAPS/50 mM Tris pH 9, then diluted with 50 μL 50 mM Tris pH 9, and applied to strong anion exchange beads (Q-ceramic HyperD F, Pall Life Sciences, Ann Arbor, MI) in a 96-well Silent Screen filtration plate (NUNC, Rochester, NY). After binding for one hour, a vacuum was applied to the sample-loaded anion exchange beads in filter plates to collect fractions. Fractions were collected (200 μL total) using buffers of decreasing pH at pH 9, 7, 5, 4, and 3 (Fx1-5, respectively). A final fraction (Fx6) was collected by treating the beads with 33.3% isopropanol/16.7% acetonitrile (ACN)/0.2% trifluoroacetic acid (TFA).

#### SELDI mass spectrometry

Aliquots of each fraction (10 μL) were added to 90 μL binding buffer and applied to ProteinChip SELDI arrays (Bio-Rad Laboratories, Hercules, CA). Binding buffers were 0.1 M sodium acetate buffer (pH 4) for CM10 arrays (a negatively-charged weak cation-exchange surface), 10% ACN/0.1% TFA for H50 arrays (a reversed-phase surface), and 0.1 M sodium phosphate, 0.5 M NaCl, pH 7 for IMAC30-Cu^++^ arrays (a histidine-, tryptophan-, and cysteine-binding surface). After washing and air-drying, sinapinic acid (Bio-Rad) was added to each ProteinChip surface (two applications of 1.0 μL of a 50% saturated solution in 50% ACN/0.5% TFA).

The discovery cohort samples were analyzed in a PBSIIc SELDI time of flight (TOF) mass spectrometer (Ciphergen, Fremont, CA). Spectra from 0–75,000 Da were acquired in positive ion mode using a single laser energy with instrument settings: optimized mass range of 3,000–30,000 Da, focus mass = 16,500 Da, detector sensitivity = 6, and deflector mass = 3,000 Da. Spectra were calibrated externally using Protein Standards II (Ciphergen): hirudin (7,033.6 Da), cytochrome c (bovine (12,231 Da)), bovine myoglobin (16,952 Da), and bovine erythrocyte carbonic anhydrase (29,024 Da).

Samples for the confirmation cohort and the protease inhibitor/validation cohort were analyzed in a newer PCS4000 SELDI mass spectrometer (Bio-Rad, Hercules, CA). Spectra were acquired at low (3200 nJ), medium (4500 nJ), and high (7250 nJ) laser energies for mass ranges of 2,500–10,000 Da (focus 5,000 Da), 5,000–30,000 Da (focus 14,000 Da), and 20,000–200,000 Da (focus 50,000 Da), respectively. Detector blanking was set to 2,000 Da for all three laser energies. Spectra were calibrated externally using the Protein Standards II (Bio-Rad) as above for low and medium laser energy spectra, and using erythrocyte carbonic anhydrase, enolase (46,6701 Da), bovine serum albumin (66,433 Da), and human IgG (147,300 Da) as calibration proteins for high laser energy spectra.

Spectra were post-processed using noise reduction and baseline subtraction algorithms in ProteinChip Data Manager 3.5 software (Bio-Rad). Batches of spectra were normalized for total signal intensity, and spectra of poor quality were discarded. Peaks were detected and quantified in batches for spectra acquired on the same day under the same conditions. The criterion for a consensus peak location was occurrence with signal-to-noise ratio (s/n) greater than 5 in at least 20% of spectra. Tables of all peaks were prepared and used for statistical analysis.

#### Statistical tests

The rank sum Mann-Whitney (MW) *U* test was employed to test the null hypothesis that the SELDI peak intensities in the KD and FC groups were not different. The tables of SELDI peak intensities therefore represent more than 1,000 tests of these hypotheses. We used global and local false discovery analyses to aid in the interpretation of the results [23,24]. The global false discovery rate (FDR) was used to control for Type I rejection of the null hypothesis, and was determined when the intensities of all the SELDI peaks were permutated 100 times [25]. The local FDR was determined when the intensities of each peak were permutated 100 times [26]. Spearman correlation analyses with FDR correction were performed [24]. Additional Pearson correlation analyses, Mann-Whitney signed rank test, and Fisher’s exact test were performed using Prism 6 software (GraphPad).

#### Analysis of correlations in SELDI data

We utilized our clustering algorithms, which cluster SELDI peaks that are biologically or technically correlated [27] or simply technical aliases [28], to evaluate the correlation of this peptide with other peaks in these data sets. Each feature of the tables of peaks was converted to mean-centered centroids and tested for correlation with the other features.

#### Identification of peak

Two strategies were used to partially purify the protein peak in order to perform trypsin digestion and peptide sequence analysis. In the first strategy, plasma (200 μL) from the discovery cohort was mixed with 300 μL of the 9 M urea buffer as described above for fractionation. The denatured plasma was then applied to 0.5 ml packed Q ceramic HyperD F beads in spin columns at pH 9, and the same buffers of decreasing pH were used to obtain fractions. Fractions containing the peak were then adjusted to 5% (v/v) ACN/0.3% TFA and adsorbed with polystyrene/divinylbenzene reverse phase beads (12–14 μm, PLRP-S (Agilent)). Fractions using increasing ACN concentrations were collected. Selected fractions were diluted with PBS and applied to a YM50 centrifugal ultrafiltration concentrator (Amicon) to remove IgG. The resulting fraction was subjected to reverse phase chromatography as above, dried, then subjected to sodium dodecylsulfate-polyacrylamide gel electrophoresis (SDS-PAGE) using 10% NuPage gels with 2-(*N*-morpholino) ethanesulfonic acid (MES) running buffer without dithiothreitol (Invitrogen). After staining with colloidal Coomassie blue and destaining, candidate bands were excised and cut into two pieces. One piece was extracted with 50% formic acid, 25% ACN, 15% isopropanol, and 10% H_2_O to locate the peak by SELDI-MS on a PCS4000 mass spectrometer; the remaining piece was subjected to in-gel trypsin digestion without reduction and alkylation. The spectra for the isolated protein were calibrated externally using the peaks for the hirudin (7,033.6 Da) and cytochrome c (bovine (12,231 Da)) calibrants. The tryptic digest was applied to NP20 ProteinChip arrays with matrix and an MS spectrum obtained using an Applied Biosystems Q-Star mass spectrometer fitted with a ProteinChip array interface (Applied Biosystems and Ciphergen). MS peaks were selected manually for analysis by tandem mass spectrometry (MS/MS) and sequenced using Mascot software (Matrix Science).

The second purification strategy utilized chromatography on Protein A-conjugated beads as the first step instead of Q anion exchange chromatography. A KD subject’s plasma from the discovery cohort (100 μL) was diluted with 200 μL phosphate buffered saline (PBS) and adsorbed to 0.05 ml Protein A-conjugated ceramic beads (Pall Corp.). After one hour of incubation, the beads were washed four times with 200 μL ml PBS, followed by two 50 μL extractions with 0.1 M acetic acid. The protein was found in the first acetic acid extract, and was further purified by reverse phase chromatography, centrifugal ultrafiltration, and SDS-PAGE as above.

#### Mass Spectrometric Immuno Assay (MSIA)

Affinity pipette tips conjugated with antibody against human serum amyloid A were prepared by Intrinsic Bioprobes of Thermo Fisher Scientific [29,30]. The affinity pipette tips were used to capture SAA from 10 μL plasma samples from the third protease inhibitor/validation cohort in duplicate. Blinded plasma samples from six KD subjects and six FC subjects from the protease inhibitor/validation cohort were adsorbed to these pipette tips using an automatic stand-mounted multi-channel pipettor (Finnpipette). After extensive washing, the tips were eluted with sinipinic acid directly onto matrix assisted laser desorption ionization (MALDI) sample plates. MALDI mass spectra were acquired using a Bruker Autoflex II spectrometer. Exported spectra were baseline subtracted, minimally smoothed, and calibrated internally using ApoC-I and SAA1 peaks as calibrants using mMass software [31], and graphed with Datagraph software (Visual Data Tools Inc.).

## Results

### Discovery Cohort

SELDI-TOF-MS on pH fractionated plasma samples from 10 KD subjects and 10 FC subjects yielded 1,638 peaks from 1,020 mass spectra (Table 2). The intensities of 274 (16.7%) peaks differed significantly (*p* < 0.05) between KD and FC subjects (Table 2), whereas the global FDR method predicted approximately 60 false discoveries at *p* < 0.05. Twenty-six peaks had a local FDR < 5% with *p* ≤ 0.0007 (Table 2).

**Table 2:** Identification of SELDI peaks differentiating KD and FC in three studies.

	*p*-value^a^	Number^b^ (%) of peaks	Local FDR
Discovery cohort	< 0.0001	2 (0.12)	≤ 0.032
	< 0.0005	15 (0.92)	≤ 0.032
	0.0007	26 (1.6)	< 0.05
	< 0.005	58 (3.5)	≤ 0.13
	< 0.01	105 (6.4)	≤ 0.14
	< 0.05	274 (16.7)	≤ 0.30
Confirmation cohort	< 0.00001	14 (0.9)	≤ 0.001
	< 0.0001	45 (3.2)	≤ 0.004
	< 0.001	104 (7.3)	≤ 0.016
	< 0.005	206 (14.5)	≤ 0.045
	0.006	229 (16.1)	< 0.05
	< 0.01	266 (18.7)	≤ 0.09
	< 0.05	439 (30.9)	≤ 0.30
Validation and protease inhibitor cohort (EDTA)	< 0.001	2 (0.1)	0.29
	< 0.005	15 (1.1)	0.29
	< 0.01	34 (2.4)	0.29
	< 0.05	174 (12.2)	≤ 0.30
Validation and protease inhibitor cohort (EDTA/PI)	< 0.001	4 (0.3)	≤ 0.14
	< 0.005	33 (2.3)	≤ 0.14
	< 0.01	63 (4.4)	≤ 0.16
	< 0.05	255 (17.8)	≤ 0.37

^a^by Mann-Whitney *U* test

^b^cumulative number of peaks at indicated *p* value, from 1641 peaks in Discovery cohort, and 1421 peaks in Confirmation and Validation cohorts.

PI, protease inhibitor

**Fig 1:**
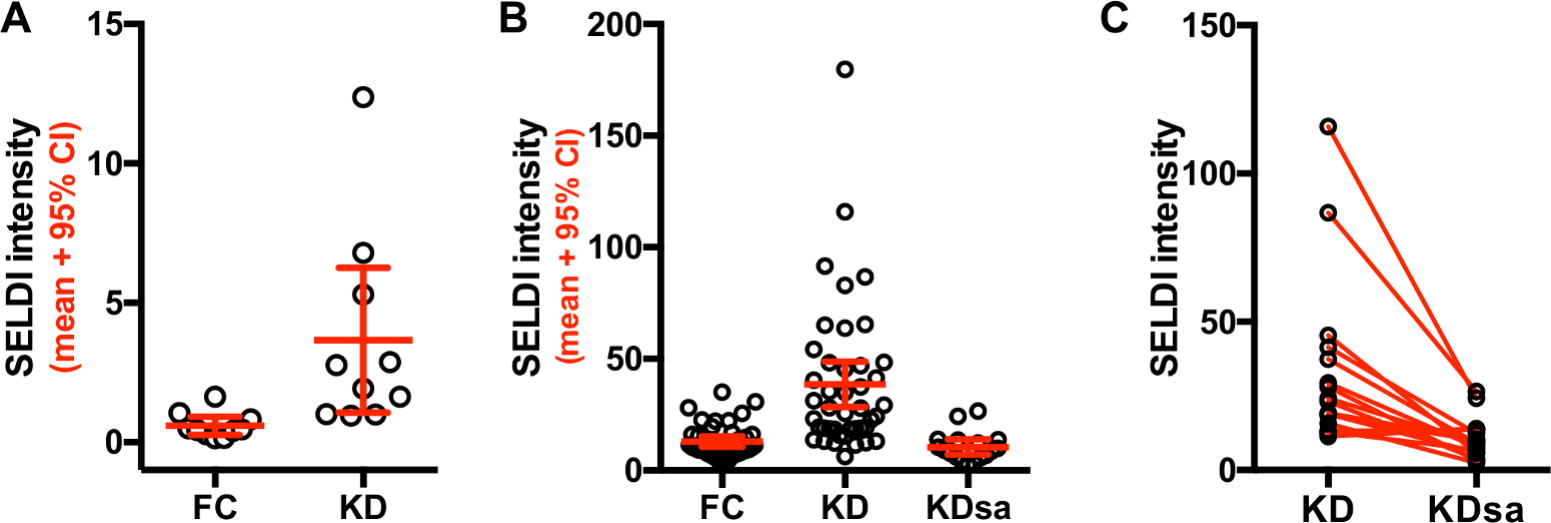
SELDI peak intensities for Fx1 CM10 7,860 Da peak in Cohort 1. A) Means and 95% confidence interval (CI) for 10 KD and 10 FC subjects in the Discovery cohort are shown. B) Mean and 95% CI for 43 KD, 42 FC and 17 KDsa from the Confirmation cohort are shown. C) SELDI intensities for the 7,860 Da peak of 17 KD subjects during their acute and subacute phases of illness, from the Confirmation cohort. The acute and subacute values for 17 KD subjects are connected with a line.

One peak of apparent mass of approximately 7,860 Da, which eluted/washed from the Q anion exchange bead at pH 9 and bound only to the negatively charged CM10 ProteinChip array, had significantly higher intensities in KD than in FC subjects (*p* = 0.0003, local FDR = 0.032). Using a criterion of s/n ≥ 2 for the presence of the 7,860 Da peak in a subject’s mass spectrum, 8 of 10 FC subjects had low intensity 7,860 Da peaks, and all 10 KD subjects had 7,860 Da peaks of higher intensity than FC subjects. Only 1 of 10 FC subjects had a peak intensity greater than the lower 95% confidence interval (CI) of the KD subjects, while 10 of 10 KD subjects had a peak intensity greater than the upper 95% CI of the FC subjects (Fig 1 and Table 3).

**Table 3:** Sequences of peptides from 7,860 Da protein.

Peptide m/z		Sequence	Position	Position
SAA1	SAA2		SAA_7860_	SAA_1-122_
2177.9		FFGHGAEDSLADQAANEWGR	35–54	86–105
2097.0		GPGGVWAAEAISDARENIQR	15–34	66–85
	1942.0	SGRDPNHFRPAGLPEKY	55–71	106–122
1914.0		SGKDPNHFRPAGLPEKY	55–71	106–122
1749.9		SGKDPNHFRPAGLPEK	55–71	106–122
	1688.8	GAEDSLADQAANKWGR	39–44	90–105
1640.8	1640.8	DPNHFRPAGLPEKY	58–71	109–122
1612.8		RGPGGVWAAEAISDAR	14–29	65–80
	1611.8	RGPGGAWAAEVISNAR	14–29	65–80
1525.7#	1525.7#	PNHFRPAGLPEKY	59–71	110–122
1456.7		GPGGVWAAEAISDAR	15–29	66–80
	1455.7	GPGGAWAAEVISNAR	15–29	66–80
821.4##	821.4##	KYFHAR	1–6	52–57
693.3	693.3	YFHAR	2–6	53–57

The SAA_7860_ peptide consists of 71 amino acids, corresponding to amino acids 52–122 of translated SAA_1-122_ (amino acids 34–104 of circulating SAA).

# D-P post-source decay

## Unique to truncated form

### Confirmation Cohort

SELDI-TOF-MS performed in duplicate for 43 acute KD, 42 FC, and 17 KDsa subjects produced 6120 mass spectra. The intensities of 439 (30.9%) of the 1,421 consensus peaks differed significantly (*p* < 0.05) between KD and FC subjects (Table 2). Sixteen percent of the peaks (229) had a local FDR < 0.05, all with *p* < 0.006.

A peak of approximate mass 7,860 Da found in the unbound Q anion exchange pH 9 fraction was again elevated in most acute KD subjects and was present at very low levels in most FC subjects (Fig 1) (*p* = 7.9 x 10^−10^, local FDR = 6.3 x 10^−7^). The SELDI intensity of this peak was greater for 33 of 43 KD subjects than the upper 95% CI of FC subjects and was greater for 4 of 42 FC subjects than the lower 95% CI of KD subjects (Fig 1).

The intensities of the 7,860 Da protein peak were compared in the 17 (of 43) KD subjects for whom acute and subacute samples were available and are shown in Fig 1C. The intensity of the 7,860 protein peaks was lower in the KDsa subjects compared to the matched acute values for these subjects. The difference between the matched values for the acute and subacute phase was statistically significant by the Wilcoxon signed-rank test (Fig 1C, *p* = 0.0002). The median intensity of the 7,860 Da protein peak in the 17 KDsa subjects was significantly lower than that of all 43 acute KD subjects (*p* = 2.7 x 10^−6^ by *t* test, local FDR = 4.6 x 10^−5^) and was not significantly different from FC subjects (*p* = 0.23, local FDR = 0.98 for FC *vs* KDsa, Fig 1B).

### Identification of the 7,860 Da Peak

**Fig 2:**
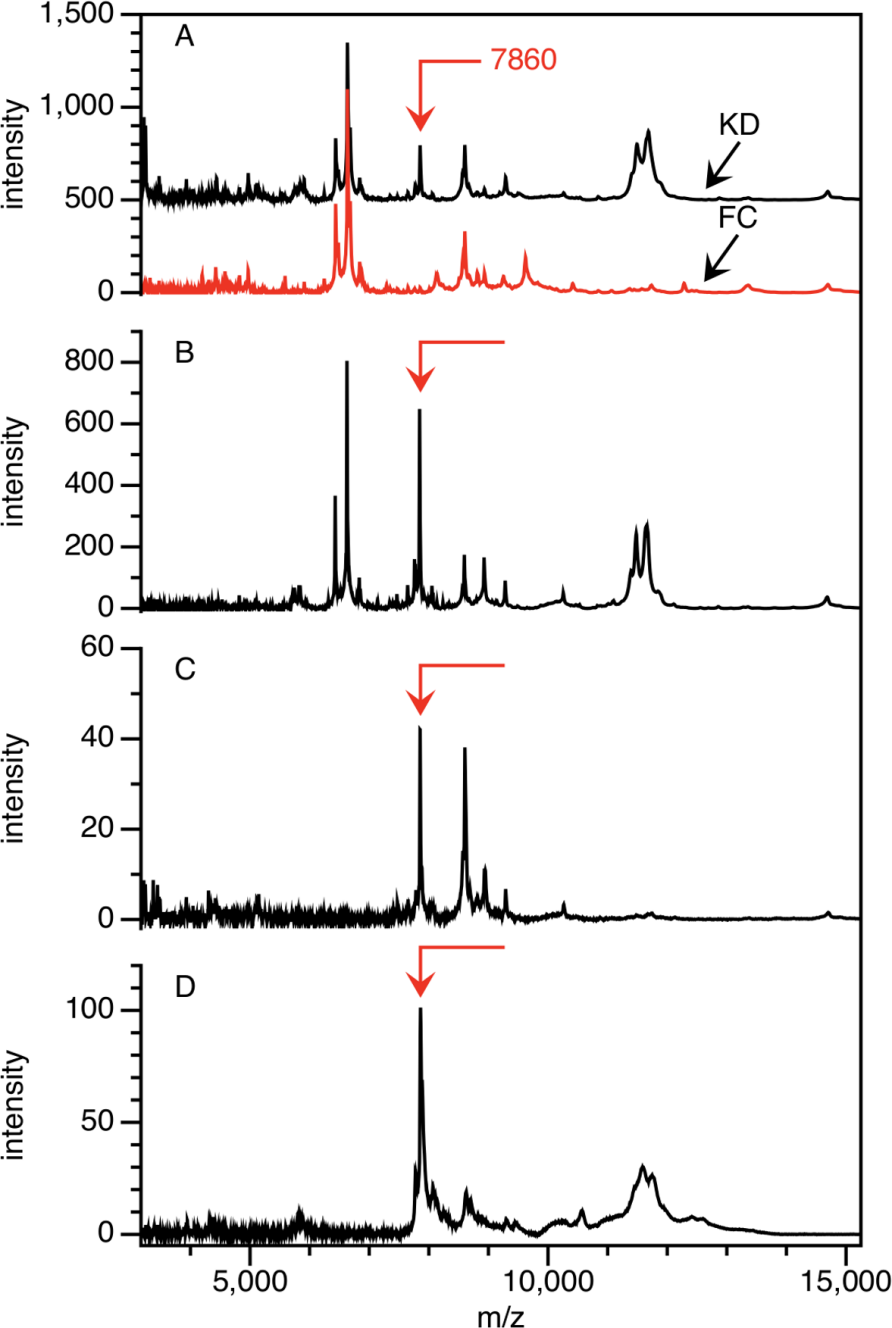
Partial purification of the 7,860 Da peak. The peak is shown at mass = 7,860 Da on CM10 ProteinChip arrays (A) in the pH 9 fraction after application of KD and FC plasma to Q anion exchange ceramic beads at pH 9; (B) in 50% ACN reverse phase fraction after application of the KD sample from (A) to PLRP beads, (C) the KD sample after using an ultrafiltration centrifugation device, and (D) the KD sample after elution from SDS-PAGE band. The FC spectrum is shown only for (A).

Our first strategy for isolating the 7,860 Da peak used the Q anion exchange fractionation method applied to plasma from a KD and FC subject with high and low levels of the peak respectively, from the discovery cohort. Additional reversed phase chromatography, centrifugal ultrafiltration and SDS-PAGE steps yielded the peak eluted from a gel band (Fig 2). The peak was confirmed to have a mass of 7,860 Da, and was not present in this FC subject. The remaining portion of the bands was digested with trypsin and analyzed by both MALDI MS and MS/MS methods.

Sequence analysis of the SDS-PAGE band containing the 7,860 Da protein revealed eight tryptic peptides and two non-tryptic peptides derived from serum amyloid A (SAA) (S1 Fig and Table 3). The processed circulating forms of SAA1 (Uniprot P0DJI8) and SAA2 (Uniprot P0DJI9) are proteins of 104 amino acids, with masses of 11,683 and 11,648 Da, respectively. All the sequenced peptides from the 7,860 Da band were aligned between amino acids 34 and 104 of circulating SAA. SAA1 and SAA2 are members of the SAA family, with 89% sequence identity for amino acids 34–104 (amino acids 52–122 of translated SAA) (Table 3). Six peptide sequences (masses 2,177.9, 2,097.0, 1,914.0, 1,749.9, 1,612.8, and 1,456.7 Da) were unique to SAA1, four (masses 1,942.0, 1,688.8, 1,611.8, and 1,455.7 Da) were unique to SAA2, and four (masses 1,640.8, 1,525.7, 821.4, and 693.3 Da) were common to SAA1 and SAA2 (Table 3).

Our second purification strategy was used for an additional KD plasma sample from the discovery cohort. Plasma was adsorbed with Protein A-conjugated ceramic beads. The 7,860 Da peak was extracted from the beads in a low pH fraction that contained eluted IgG. After further enrichment as described in **Methods**, the preparation was subjected to SDS-PAGE and trypsin digestion of candidate gel bands. Again, the sequenced peptides for the 7,860 Da peak aligned with amino acids 34–104 of circulating SAA. One non-tryptic peptide, corresponding to the 821 Da peptide ion peak, would be unique to a truncated form of SAA (amino acids 34–39, KYFHAR) whose first amino acid K was a missed trypsin cleavage site (S1 Fig lower spectrum, Table 3).

### Validation Cohort: Validation of 7,860 Da Peak as SAA

**Fig 3:**
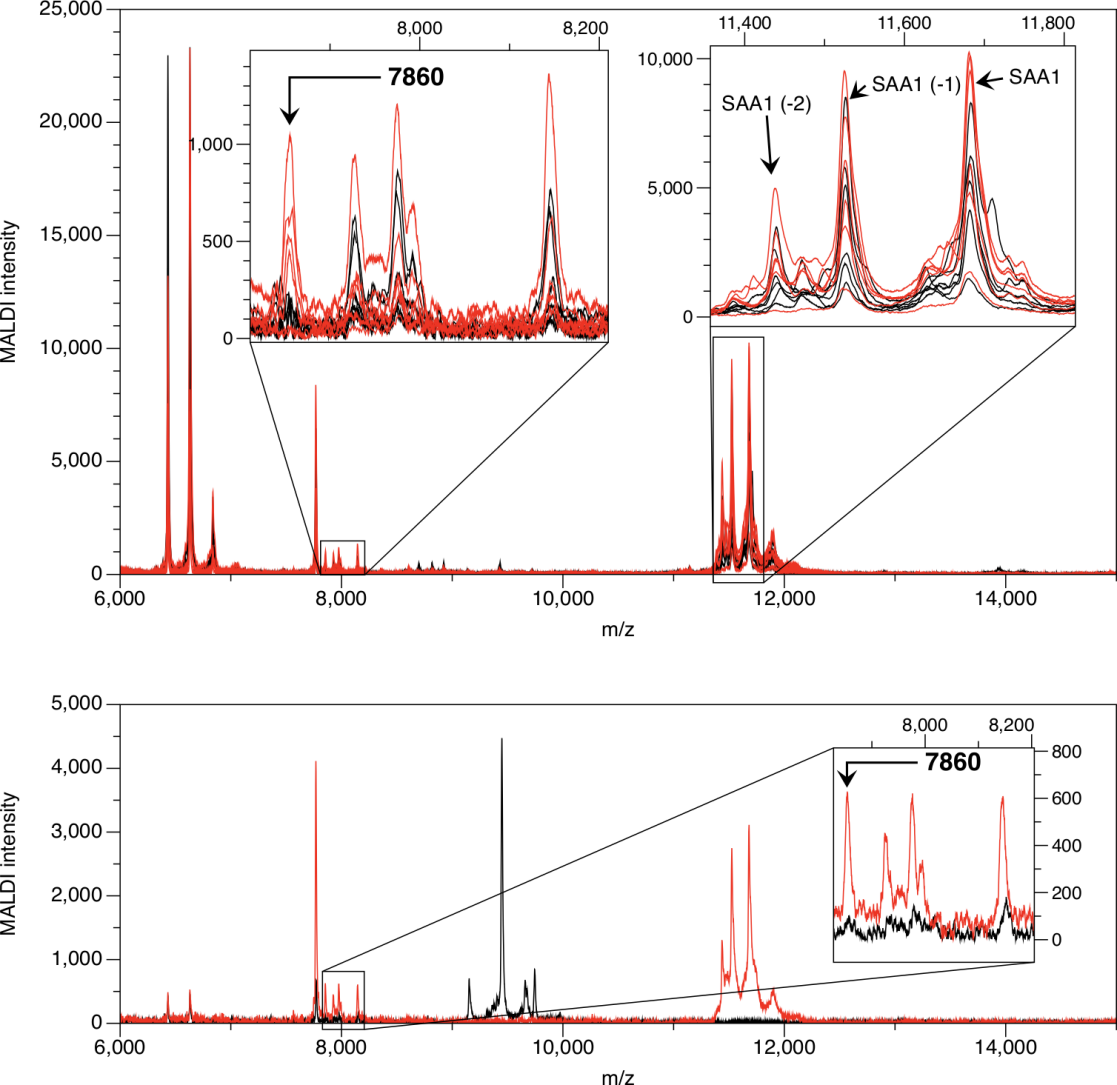
MSIA for SAA. Upper spectra: the SAA_7860_ peaks are shown in the expanded inset of 7,800–8,200 Da on the left, and circulating SAA1 and SAA2 are shown in the expanded inset of 11,400–11,800 Da on the right. The averaged spectra for replicate samples are red for KD subjects and black for FC subjects. Lower spectra: MSIA using anti-SAA IgG_1_ in averaged replicate spectra of a KD subject are shown in red, and MSIA spectra of the same KD subject using an irrelevant mouse IgG_1_ antibody are shown in black. The SAA_7860_ peak is shown in the expanded inset of 7,800–8,200 Da. The irrelevant IgG_1_ does not detect either the circulating form of SAA or the SAA_7860_ peptide.

**Fig 4:**
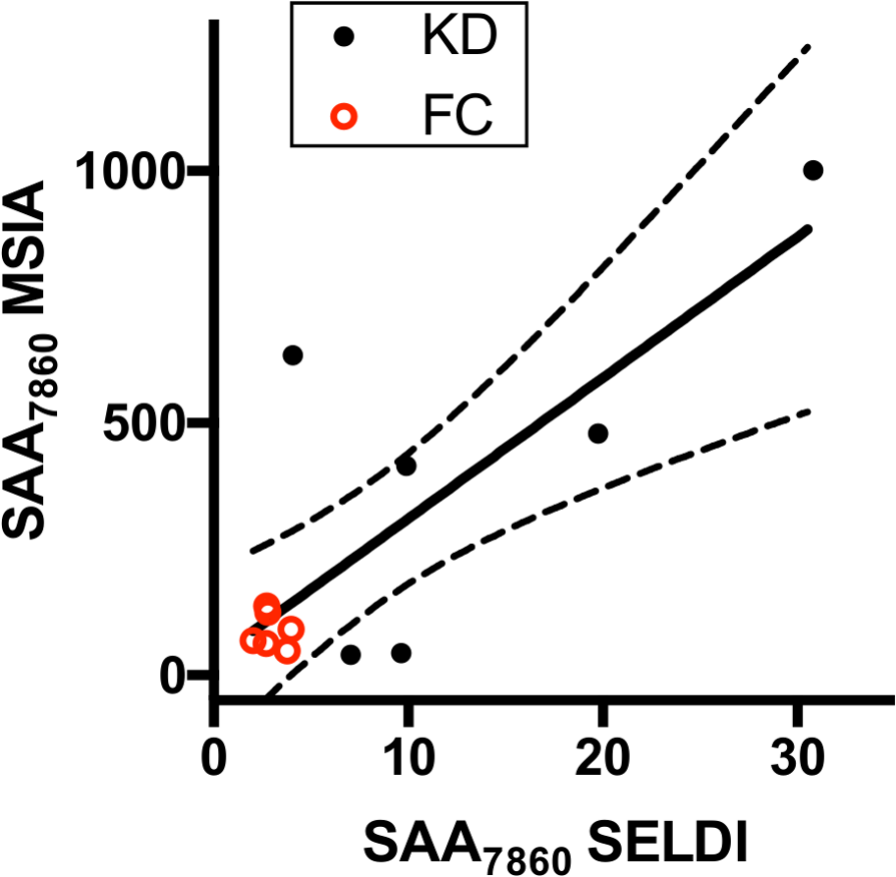
Correlation of MSIA and SELDI peak intensities. The averaged MSIA peak intensities for SAA_7860_ are plotted against the averaged SELDI peak intensities obtained during discovery studies. KD subjects are shown as solid black circles, and FC subjects are shown as open red circles. The linear regression line (solid line) with 95% CI (dotted lines) is shown.

We used the Mass Spectrum Immuno Assay (MSIA) hybrid technique to confirm the identification of the m/z 7,860 peak as a truncated form of SAA (SAA_7860_). Anti-SAA was covalently linked to a glass support inside pipette tips as described in **Materials and Methods**. Proteins were eluted directly onto a MALDI sample plate and analyzed by MALDI mass spectrometry (Fig 3). Peaks of high intensity for the circulating forms of SAA1 (SAA1_19-122_), SAA1_20-122_, and SAA1_21-122_ were found at masses 11,683.7, 11,526.5, and 11,439.4 Da, respectively in both KD and FC subjects, as well as peaks representing SAA2 (SAA2_19-122_, SAA2_20-122_, and SAA2_21-122_ at masses 11,647.8, 11,491.6, and 11,404.5 Da, respectively) in some KD and FC subjects. Peaks of lower intensity at lower m/z ratios also appeared in eluates from the anti-SAA MSIA tips. Peaks corresponding to the SAA_7860_ fragment were present in plasma of all of the six KD subjects and in none of the six FC subjects. The SELDI peak intensities from the fractionated plasma of these subjects correlated positively with the SAA MSIA intensities (Pearson r = 0.79, *p* = 0.002)(Fig 4).

### Validation Cohort: Protease Inhibition

The presence of the SAA_7860_ fragment in plasma samples raised questions about whether it was present in the subject or was the result of proteolytic cleavage *ex vivo* after sample collection. A third cohort was designed to test the hypothesis that the SAA_7860_ fragment was formed as a result of *ex vivo* proteolysis after collection. In this cohort, blood was collected at diagnosis in standard EDTA tubes and in commercially available EDTA tubes containing a cocktail of protease inhibitors (EDTA/PI). Samples were collected from 15 acute phase KD subjects and 9 FC subjects. Plasma samples from both types of tubes (EDTA and EDTA/PI) were fractionated at the same time and analyzed by SELDI mass spectrometry exactly as in the second confirmation cohort with the same laser energies, ProteinChip arrays, calibrations, and consensus 1,421 peak locations.

**Fig 5:**
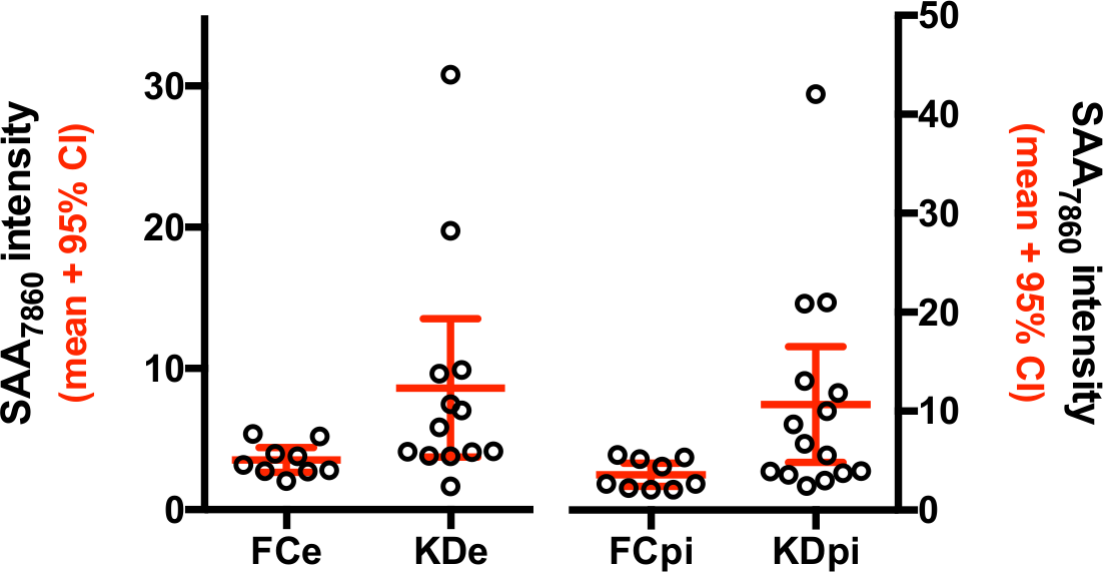
SELDI peak intensities for SAA_7860_ peptide in the Validation cohort. The mean and 95% CI for the SELDI intensities of the SAA_7860_ in 15 KD and 9 FC subjects are shown for EDTA (FCe and KDe, left axis) and protease inhibitor (FCpi and KDpi) plasma (right axis).

The SAA_7860_ peak was found in the pH 9 fraction of the EDTA and EDTA/PI plasma of KD subjects, thus excluding post-collection proteolytic generation of this peptide fragment. In plasma from both types of tubes, the median intensity of the SAA_7860_ peak was greater in the KD subjects than in the FC subjects (Fig 5). The EDTA vs EDTA/PI values for the KD subjects were not different by unpaired *t* test (S2 Fig).

### Relationship to Full-Length SAA or Other Protein Peaks

We analyzed whether full-length SAA_19-122_ discriminates KD subjects from FC subjects. The SELDI peak intensities of neither full length SAA2_19-122_ (pH 9 fraction) nor full length SAA1_19-122_ (pH 4 fraction) differed between KD and FC subjects (S3 Fig). The SELDI intensities for full-length SAA2 and SAA1 were significantly lower in KDsa subjects than KD or FC subjects. The SELDI intensity of the SAA_7860_ protein did not correlate with the intensity of full-length SAA (S3 Fig) using the Spearman correlation test.

Among the software algorithms we used to evaluate correlations in complex sets of SELDI biomarker discovery studies, our Technical Alias algorithm causes SELDI peaks to cluster when they are technical aliases of one another (*e*.*g*., the same protein found in different fractions, or the same protein with different charges) [28]. The Technical Alias clustering analysis performed on the confirmation cohort data indicated that the SAA_7860_ peaks from low and medium laser energy of the pH 9 fraction and CM10 ProteinChip array formed a cluster. These peaks did not cluster with peaks of similar m/z ratio from any other chromatographic fractions or SELDI surfaces, nor did they cluster with other peaks of different apparent mass, indicating different charges of the same species (*e*.*g*., when z = 2, or when a peak represents a doublet of the protein with a single charge). When the clustering algorithm was not restricted to identification of aliases (the Angle Vector Quantization/Bayes Information Criterion method [27]), the SAA_7860_ peaks from low and medium laser formed a cluster, but did not cluster with any of the other 1,419 peaks in the confirmation cohort samples (data not shown). Thus the SAA_7860_ peak did not correlate with any of other protein peaks.

### Correlation with Laboratory and Clinical Evaluations

**Fig 6:**
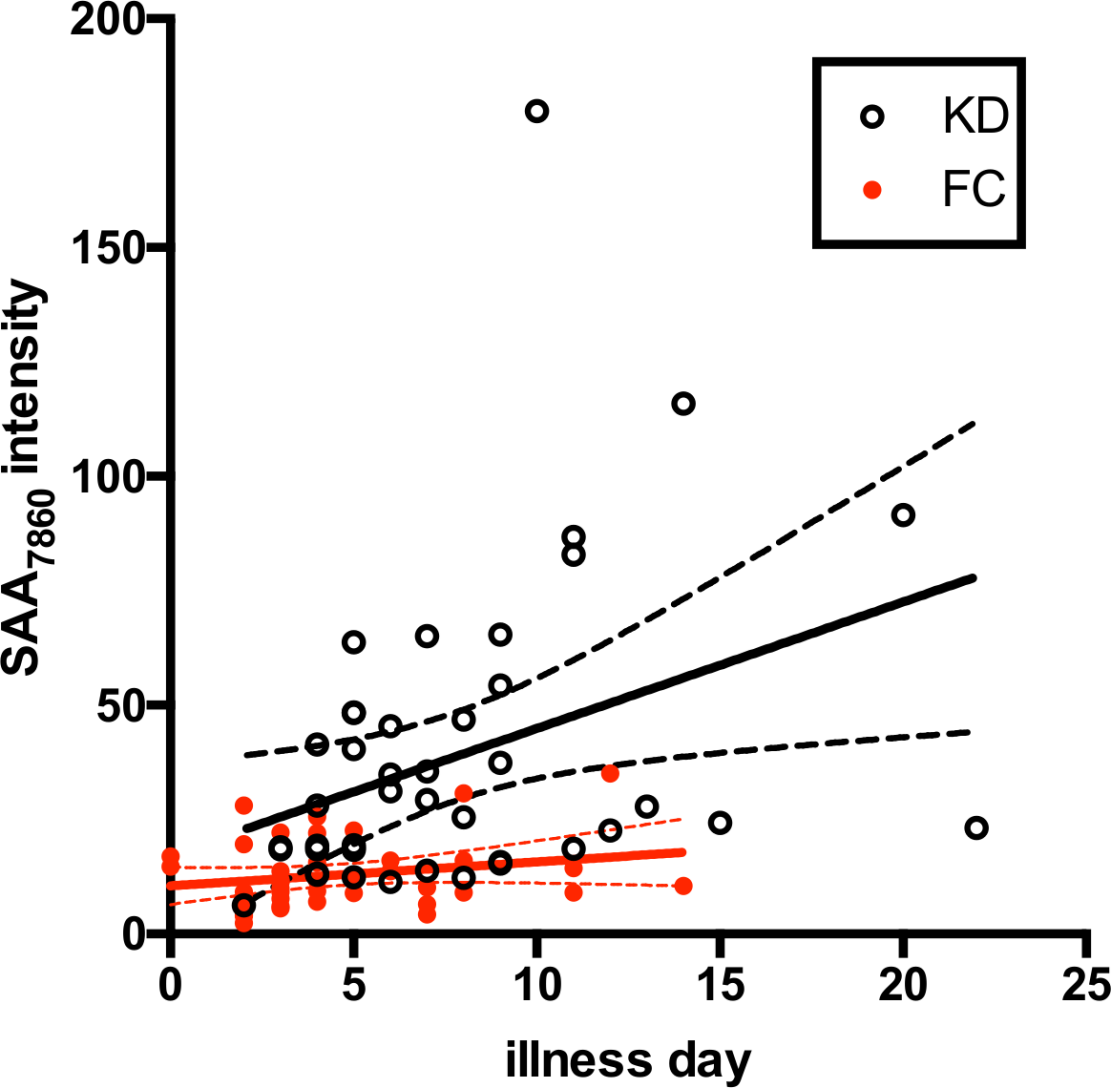
Correlation of Days of Illness at time of study vs SAA_7860_ peptide peak intensity. SELDI SAA_7860_ peak intensities are plotted versus days of illness at the time of study for KD and FC subjects in Cohort 2. KD, open circles; FC, closed red circles. Linear estimate lines with 95% CI are shown in black for KD and red for FC subjects.

The SAA_7860_ peak intensities did not correlate with acute CRP, ESR, GGT, or white blood cell count (WBC) in any of the studies. SAA_7860_ peak intensities had weak positive correlations with platelet count in KD and FC subjects in the confirmation cohort only (Table 4 and S4 Fig). There was no apparent relationship between the quantity of the SAA_7860_ peptide and IVIG response or coronary artery status in the KD subjects (data not shown). The SAA_7860_ SELDI intensities correlated positively with days of illness at diagnosis for KD subjects (*p* = 0.0055 for the Pearson correlation test) but not for FC subjects (Fig 6).

**Table 4:** Correlations of SAA_7860_ peptide with clinical laboratory test results in confirmation cohort.

		Spearman		
SAA_7860_ vs	group	*p* value	local FDR	Corr
ESR	KD	NS	NA	NA
	FC	0.004	0.007	0.546
Platelet count	KD	0.034	0.136	0.324
	FC	0.012	0.016	0.400
WBC count	KD	NS	NA	NA
	FC	1.3 x 10^−4^	2.6 x 10^−4^	0.575

NS = Not significant at *p* > 0.05; NA = Not applicable at *p* > 0.05.

Correlation between SAA_7860_ and CRP, ESR, GGT, Platelet count, and WBC count were evaluated for the discovery, confirmation and validation cohorts (summaries of these clinical test results are in Table 1). Only the positive correlations for the second confirmation cohort are shown in the table. There were no significant correlations for of the SAA_7860_ peptide with CRP or GGT in the confirmation cohort, and there were no significant correlations between SAA_7860_ and any of the clinical tests in the discovery or validation cohorts.

## Discussion

In the absence of a specific diagnostic test for KD, approximately 15% of children receive their diagnoses more than than 10 days after symptom onset [11], leading to a nearly threefold increased risk of coronary aneurysms [10]. We used SELDI-TOF-MS to screen the plasma proteome for candidate KD biomarkers. We used anion exchange chromatography to fractionate the plasma for this study in order to identify less abundant peptides and proteins, and used a top-down approach that analyzed the samples without prior treatment with proteases. This technique permitted us to find differences in the SAA peptide.

In our three cohorts of acute-phase KD subjects, numerous SELDI-TOF-MS peaks differed significantly in the plasma of KD vs FC subjects. However one protein peak was particularly different in all three cohorts in distinguishing KD subjects from FC and subacute KD subjects. This peak was elevated in most KD subjects but was low or undetectable in FC subjects. The purified peak had a mass of 7,860 Da and was identified as a form of SAA truncated at lys-34 that was not an artifact of *ex vivo* proteolytic digestion.

We utilized the hybrid MSIA technique to confirm the identity of the SAA_7860_ peptide as a fragment of SAA and to validate the quantitative peak intensities obtained by SELDI. The MSIA technique has the ability to identify some post-translational variants of plasma proteins and has been used to identify the presence of full-length and truncated forms of SAA in plasma [29]. An additional MSIA assay assessed the specificity of the anti-SAA monoclonal antibody. An irrelevant mouse IgG_1_ did not detect either full-length SAA or the SAA_7860_ peptide in the MSIA system. A conventional ELISA technique would not have detected the SAA_7860_ peptide, since its signal would have been a minor contributor to the overall SAA content of the plasma.

In a large study of children with acute viral illnesses, Miwata *et al*. found that SAA was elevated in several illnesses compared to normal individuals, or the same patients after convalescence [32]. In that study, eight patients with KD had elevated levels of SAA, even higher than the patients with acute viral illnesses such as measles. SAA levels in these KD subjects also decreased significantly 14–26 days after the onset of illness, as it did for the patients with viral illnesses. Subsequent studies showed that SAA in KD plasma is predominantly associated with HDL-like particles in plasma, possibly replacing ApoA-I[12]. Comparative SAA levels in KD and FC subjects were not determined in that study, but 15 of 17 FC subjects were positive for SAA by Western blotting, while all acute KD subjects were positive for SAA. SAA was undetectable in all KD subjects during the subacute phase. In our study, the SELDI intensity of SAA1 and SAA2 were not significantly different between KD and FC subjects, nor did these intensities correlate with the SAA_7860_ peptide.

Examples of other post-translationally modified proteins that are biomarkers in plasma include the previously identified slightly truncated forms of SAA [29], ApoA-I, and ApoA-II [33], the glutathionylated and truncated forms of transthyretin in ovarian cancer [34] and in a rat model of glioma growth [35], and truncated forms of ITIH4 in ovarian cancer [34].

Amyloid protein A is a component of fibrillar amyloid deposits in conditions such as rheumatoid arthritis. Amyloid protein A was first identified as a proteolytic derivative of SAA after sequence analysis of the protein purified from amyloid deposits [36]. Amyloid protein A is a 76 aa N-terminal derivative of SAA (aa 19–94 of translated SAA). Subsequent studies have demonstrated that cathepsin B can generate amyloid protein A from purified SAA [37]. The proteolytic fragments generated following treatment of SAA with cathepsins B, D and L, matrix metalloproteinases 1, 2, and 3, and elastase have been documented [38–40]. The SAA_7860_ peptide is not seen in any of the mass spectra from these studies.

We were unable to find description of a SELDI peak of this m/z ratio in any published study of unfractionated or fractionated human plasma or serum. Thus we propose that the SAA_7860_ peptide is a novel, previously unreported truncation of SAA. The peak intensity of the SAA_7860_ peptide in KD subjects was lower than the full-length SAA in the fractionated plasma of discovery, confirmation, and protease inhibitor/validation cohorts, and in the MSIA identity validation study. The SAA_7860_ peptide was not detectable in SELDI spectra of unfractionated plasma (data not shown). Thus the peptide would not have been discovered without a plasma fractionation procedure coupled with a mass spectrometry assay, particularly since the peak intensities of the full-length SAA and other truncated forms of SAA are much higher than the SAA_7860_ peptide.

The N-terminus of the SAA_7860_ peptide appears to correspond to lys-34 of SAA. An enzyme with specificity similar to Peptidyl-lys metalloendopeptidase (LysN) could cleave the asp-33/lys-34 peptide bond common to SAA1 and SAA2 (http://web.expasy.org/peptide_cutter/peptidecutter_enzymes.html#LysN). An enzyme of human origin with this specificity has not been reported, and LysN is found in certain fungi, including *Grifola frondosa* and *Armillaria mellea*. However the asp-33/lys-34 peptide bond is not the only possible proteolytic cleavage site necessary for the appearance of SAA_7860_. Aminopeptidases might cleave successive N-terminal amino acids until the eventual appearance of the SAA_7860_ peptide, similar to the appearance of SAA1_20-122_, SAA1_21-122_, and SAA1_22-122_ from full length SAA1_19-122_ found in plasma, and seen in our MSIA spectra. The source (human or pathogen) and specificity of the enzymatic activity may be informative of the pathophysiology of KD.

We recognize both strengths and limitations to our study. Novel proteomic methods were used in successive discovery, confirmation, and validation cohorts. However the total number of subjects studied remains small. Although subject classification was performed by a pediatric infectious disease specialist highly experienced in the diagnosis of KD (JCB), without a gold standard test there is always the possibility that subjects could have been incorrectly assigned to the KD or FC cohorts. Because the SELDI peak intensity of this peptide does not correlate with most clinical parameters that distinguish KD from FC subjects, it has the potential to be useful in the development of diagnostic algorithms for KD [41] in combination with other markers. However the need for sophisticated sample preparation and SELDI techniques will limit the development of this method for assessing the SAA_7860_ peptide as a point-of-service diagnostic test. We are examining other techniques that could distinguish the proteolytic fragment from the parent molecule. Techniques such as immunoprecipitation followed by western blot analysis might be effective, but would not be optimal for a point-of-service technique. Antibodies that can detect the truncation cleavage site, or aptamer-based assays could be effective point-of-service techniques. It will also be interesting to determine in future studies whether the SAA_7860_ peptide is elevated in other types of pediatric vasculitis [42].

Using SELDI-TOF-MS to identify biomarkers for the diagnosis of KD, we detected a novel SAA_7860_ peptide at higher levels in the plasma of acute KD than in subacute KD and FC subjects. Future studies will examine the enzymatic pathways that might generate such a fragment and the role of this peptide in disease pathogenesis.

## Supporting Information

S1 Fig MS peaks of trypsinized 7,860 Da peptide. (A) MS peaks (m/z 1,100–2,650) eluted from trypsin-digested band from Fig 2D. Red boxes highlight the peptide ion peaks corresponding to tryptic fragments of SAA1 or SAA2, and the green box highlights the peptide ion peaks corresponding to other non-tryptic fragments of SAA1 or SAA2. (B) MS peaks (m/z 670–875) eluted from an SDS-PAGE band from another patient. The red box highlights the peptide ion peak (mass = 693 Da) that corresponds to a tryptic fragment of SAA1 or SAA2, and the green box highlights the peptide ion peak (mass = 821 Da) that corresponds to a non-tryptic fragment.
(PDF)

S2 Fig SAA_7860_ in KD subjects. SELDI intensities of SAA_7860_ in EDTA and PI tubes from KD subjects in Cohort 3 (median plus IQR).
(PDF)

S3 Fig SELDI intensities for SAA_7860_ peptide, full-length SAA1 and sull-length SAA2. The SAA_7860_ peptide and full length SAA2 peak intensities from the pH 9 Q fraction from the confirmation cohort are shown in (A) and (B), respectively, and full length SAA1 peak intensities from the pH 4 Q fraction in (C). The error bars depict the median and IQR for the samples from the confirmation cohort. SELDI intensities of the SAA_7860_ peptide from patients in the confirmation cohort are plotted versus SAA2 (D) and SAA1 (E). The correlation was not significant when compared as KD subjects only or KD plus FC subject combined. KD subjects are shown as black circles, FC subjects are shown as open red circles.
(PDF)

S4 Fig Correlation plots of clinical parameters vs SAA_7860_ peptide. Correlation plots are shown for the SAA_7860_ peptide *vs* ESR (A), platelet count (B), and WBC count (C) for KD and FC subjects in the confirmation cohort. KD subjects are shown as black circles, FC subjects are shown as open red circles. (A) ESR correlated with SAA_7860_ only for FC subjects (red lines). (B) SAA_7860_ correlated with platelet count in both KD (black lines) and FC (red lines) subjects. (C) SAA_7860_ correlated with WBC count only in FC subjects (red lines). Solid and dashed lines indicate linear regression estimate with 95% CI, respectively.
(PDF)

S1 File SELDI intensities and clinical laboratory values. The SELDI intensities and available clinical laboratory values evaluated in the manuscript are present in spreadsheet form for the three cohorts of subjects.
(XLSX)
