# Supplementary figures and images for: A Novel Truncated Form of Serum Amyloid A in Kawasaki Disease
Source: PLoS One. 2016 Jun 6;11(6):e0157024. doi: 10.1371/journal.pone.0157024 (PMC4894573; doi:10.1371/journal.pone.0157024)

**A**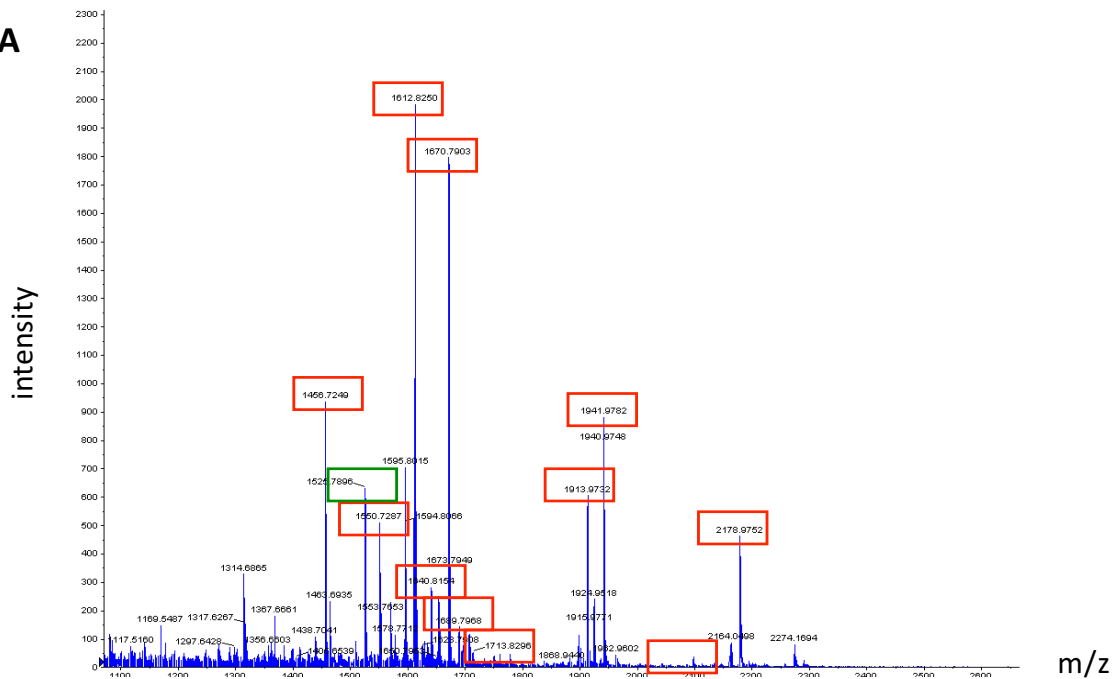**B**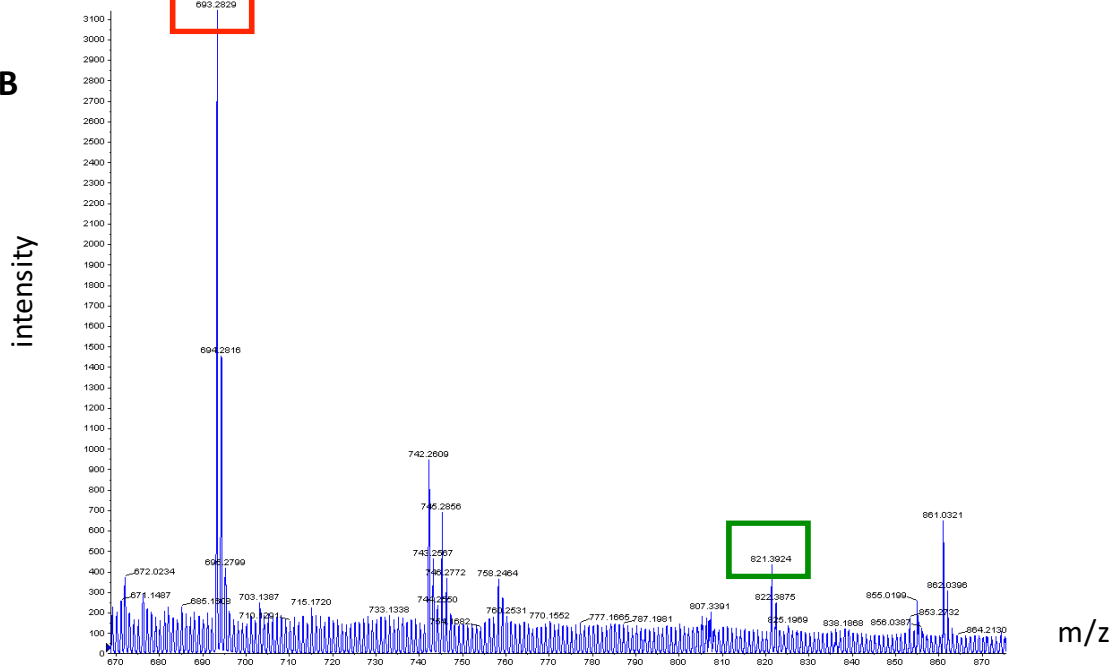

Supplement: S1 Fig — (A) MS peaks (m/z 1,100–2,650) eluted from trypsin-digested band from Fig 2D. Red boxes highlight the peptide ion peaks corresponding to tryptic fragments of SAA1 or SAA2, and the green box highlights the peptide ion peaks corresponding to other non-tryptic fragments of SAA1 or SAA2. (B) MS peaks (m/z 670–875) eluted from an SDS-PAGE band from another patient. The red box highlights the peptide ion peak (mass = 693 Da) that corresponds to a tryptic fragment of SAA1 or SAA2, and the green box highlights the peptide ion peak (mass = 821 Da) that corresponds to a non-tryptic fragment. (PDF) [file pone.0157024.s001.pdf]

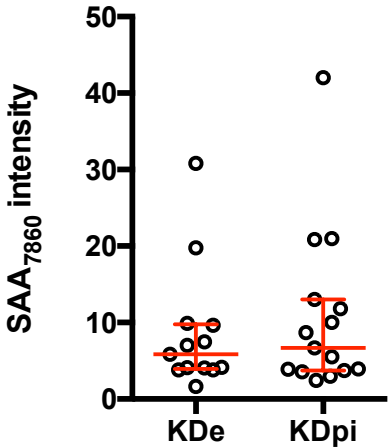

Supplement: S2 Fig — SELDI intensities of SAA7860 in EDTA and PI tubes from KD subjects in Cohort 3 (median plus IQR). (PDF) [file pone.0157024.s002.pdf]

**A**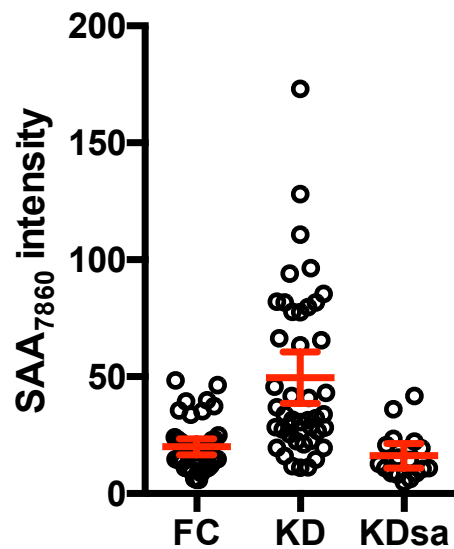**B**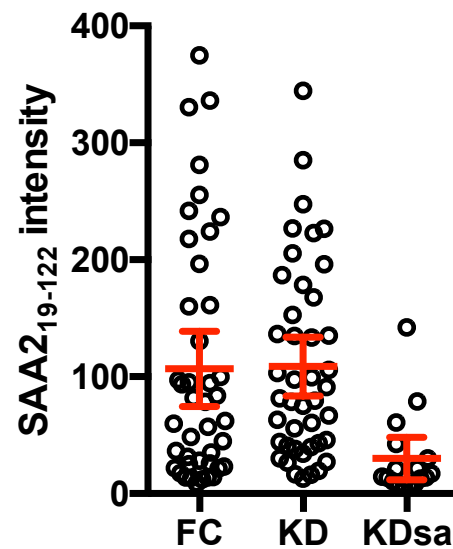**C**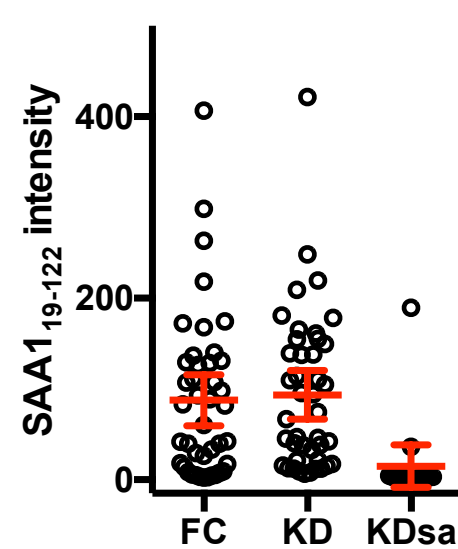**D**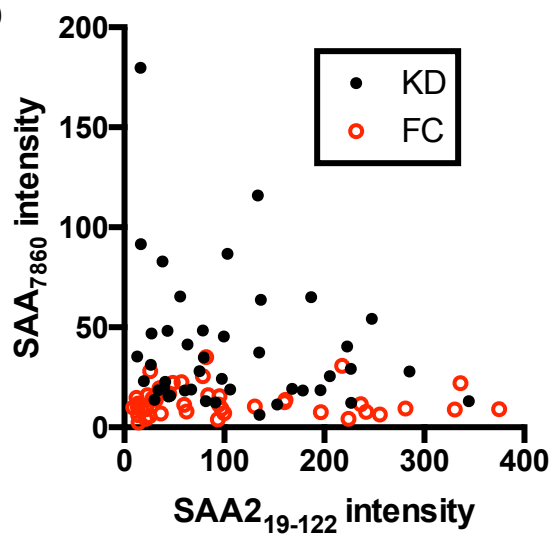**E**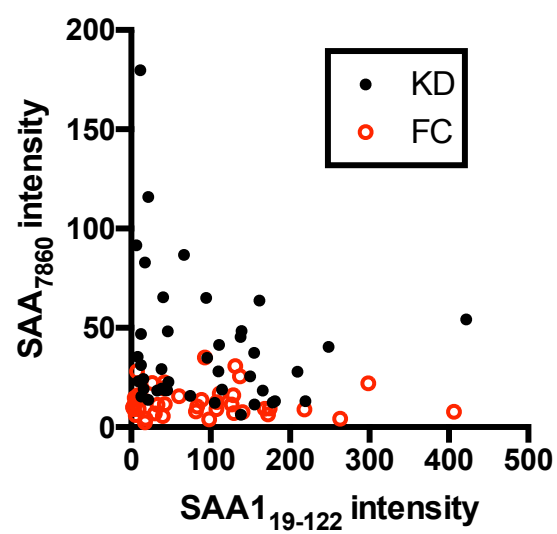

Supplement: S3 Fig — The SAA7860 peptide and full length SAA2 peak intensities from the pH 9 Q fraction from the confirmation cohort are shown in (A) and (B), respectively, and full length SAA1 peak intensities from the pH 4 Q fraction in (C). The error bars depict the median and IQR for the samples from the confirmation cohort. SELDI intensities of the SAA7860 peptide from patients in the confirmation cohort are plotted versus SAA2 (D) and SAA1 (E). The correlation was not significant when compared as KD subjects only or KD plus FC subject combined. KD subjects are shown as black circles, FC subjects are shown as open red circles. (PDF) [file pone.0157024.s003.pdf]

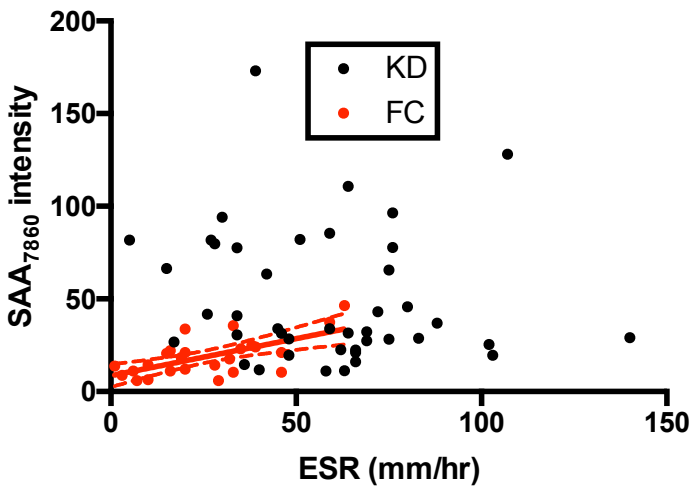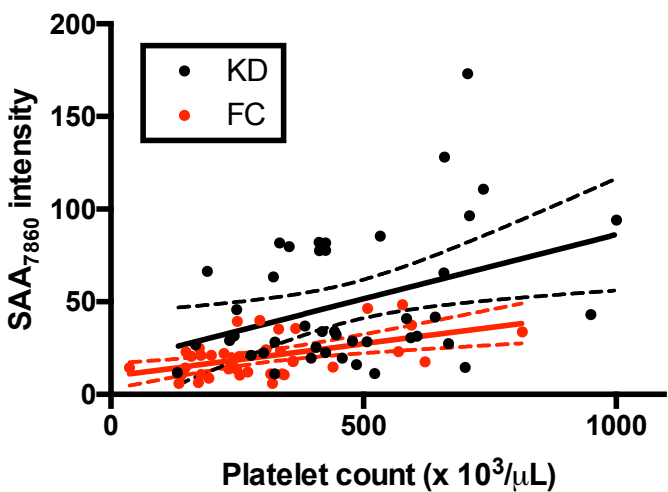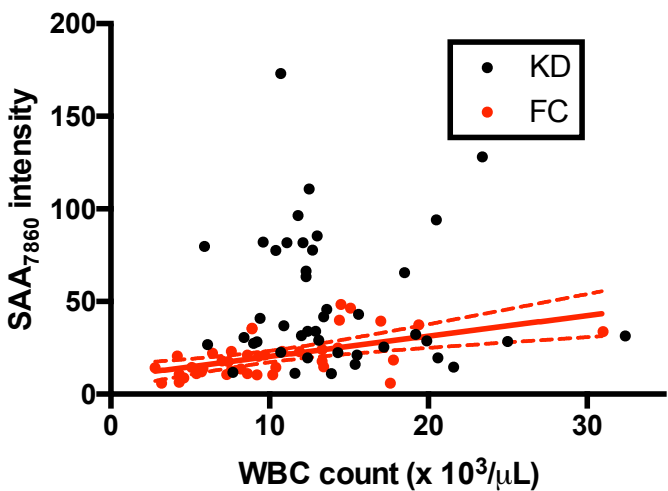

Supplement: S4 Fig — Correlation plots are shown for the SAA7860 peptide vs ESR (A), platelet count (B), and WBC count (C) for KD and FC subjects in the confirmation cohort. KD subjects are shown as black circles, FC subjects are shown as open red circles. (A) ESR correlated with SAA7860 only for FC subjects (red lines). (B) SAA7860 correlated with platelet count in both KD (black lines) and FC (red lines) subjects. (C) SAA7860 correlated with WBC count only in FC subjects (red lines). Solid and dashed lines indicate linear regression estimate with 95% CI, respectively. (PDF) [file pone.0157024.s004.pdf]
